# Production of a newly discovered PHA family member with an isobutyrate-fed enrichment culture

**DOI:** 10.1007/s00253-021-11742-9

**Published:** 2022-01-05

**Authors:** Chris M. Vermeer, Larissa J. Bons, Robbert Kleerebezem

**Affiliations:** Department of Biotechnology, Delft University of Technology, Van der Maasweg 9, 2629 HZ Delft, The Netherlands

**Keywords:** Isobutyrate, Microbial enrichment cultures, Polyhydroxyalkanoates, Poly(3-hydroxyisobutyrate)

## Abstract

**Abstract:**

Using microbial enrichment cultures for the production of waste-derived polyhydroxyalkanoates (PHAs) is a promising technology to recover secondary resources. Volatile fatty acids (VFAs) form the preferred substrate for PHA production. Isobutyrate is a VFA appearing in multiple waste valorization routes, such as anaerobic fermentation, chain elongation, and microbial electrosynthesis, but has never been assessed individually on its PHA production potential. This research investigates isobutyrate as sole carbon source for a microbial enrichment culture in comparison to its structural isomer butyrate. The results reveal that the enrichment of isobutyrate has a very distinct character regarding microbial community development, PHA productivity, and even PHA composition. Although butyrate is a superior substrate in almost every aspect, this research shows that isobutyrate-rich waste streams have a noteworthy PHA-producing potential. The main finding is that the dominant microorganism, a *Comamonas* sp., is linked to the production of a unique PHA family member, poly(3-hydroxyisobutyrate) (PHiB), up to 37% of the cell dry weight. This is the first scientific report identifying microbial PHiB production, demonstrating that mixed microbial communities can be a powerful tool for discovery of new metabolic pathways and new types of polymers.

**Key points:**

• *PHiB production is a successful storage strategy in an isobutyrate-fed SBR*

• *Isomers isobutyrate and butyrate reveal a very distinct PHA production behavior*

• *Enrichments can be a tool for discovery of new metabolic pathways and polymers*

**Graphical abstract:**

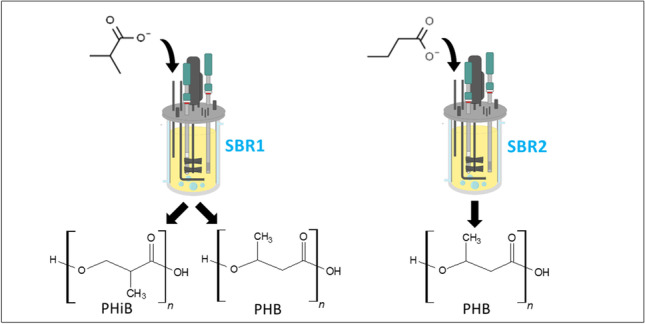

**Supplementary Information:**

The online version contains supplementary material available at 10.1007/s00253-021-11742-9.

## Introduction

Polyhydroxyalkanoate (PHA) has attracted widespread attention as an alternative to petrochemical-based plastics (Lee 1996). PHA is completely biodegradable and biobased, and has thermoplastic properties. A broad range of bacteria are able to produce this biopolymer as an intracellular carbon and energy storage (Steinbüchel 1991). The type of PHA monomer produced is determined by the substrate provided, the environmental conditions, and the microorganism, and in its turn will determine the physicochemical properties of the final polymer product. Currently, more than 150 different monomer units have been reported. Here, the vast majority is produced with metabolically engineered microorganisms or with substrates uncommon in the natural environment (Steinbüchel and Valentin 1995; Kumar and Kim 2018; Zheng et al. 2020).

An opportunity to produce PHA cost-effectively is by using mixed microbial communities and organic waste streams as feedstock. These technologies diminish the relatively large costs for sterilization and raw substrates (Kleerebezem and van Loosdrecht 2007), and as a consequence, avoid part of the waste disposal expenses (Fernández-Dacosta et al. 2015). To date, 19 pilot projects, using industrial or municipal organic waste streams as feedstock, have been operated (Estévez-Alonso et al. 2021). Here, the most common type of PHA produced is the copolymer poly(3-hydroxybutyrate-*co*-3-hydroxyvalerate) (PHBV).

A three-step bioprocess is typically used for PHA production from organic waste streams. In the first step, the heterogeneous feedstock is fermented anaerobically, and primarily converted into volatile fatty acids (VFAs), ranging from acetate to hexanoate (Serafim et al. 2008; Kleerebezem et al. 2015). These VFAs form the preferred substrate for PHA production. In a second step, a microbial community is aerobically enriched with high PHA productivity by applying feast-famine conditions. This intermittent substrate feeding strategy generates a competitive advantage for bacteria that store PHA as reservoir of carbon and electrons inside their cell. In a successive accumulation step, the PHA content of the obtained enrichment can be maximized (Reis et al. 2003; Kourmentza et al. 2017).

Under laboratory conditions, the most abundant VFAs (acetate, propionate, butyrate) of fermented waste streams have been individually assessed on their PHA production potential (Lemos et al. 2006; Jiang et al. 2011a; Marang et al. 2013). These studies reported microbial enrichments with high PHA production rates and were able to accumulate PHA up to 90% of the cell dry weight (Johnson et al. 2009a; Jiang et al. 2011a). Butyrate appeared to be the preferred VFA for PHA production, having the highest carbon uptake rate and the highest PHA yield, resulting in the highest PHA production rate (Marang et al. 2013). These laboratory studies in combination with modelling studies have resulted in an extensive understanding of the underlying PHA metabolism (Van Aalst-Van Leeuwen et al. 1997; Dias et al. 2005; Johnson et al. 2009b).

Besides linear volatile fatty acids, branched isomers like isobutyrate and isovalerate are regularly encountered products of anaerobic fermentations fed with organic waste (Dionisi et al. 2005; Mulders et al. 2020). For example, Mechichi and Sayadi (2005) show that isobutyrate can constitute up to 12 wt% of total VFA stream when fermenting olive mill wastewater anaerobically. A possible contribution to the presence of isobutyrate is the activity of proteolytic anaerobic bacteria degrading valine (Tholozan et al. 1988). The other, much more prevalent process is the isomerization of butyrate, which has been reported by multiple studies (Lovley and Klug 1982; Tholozan et al. 1988; Angelidaki and Ahring 1995). Although the precise ecological function needs to be elucidated, it is hypothesized that isomerization reduces the inhibitory effect of butyrate (Chen et al. 2017).

Despite its presence in the VFA stream of anaerobic fermentations, the PHA producing potential of isobutyrate has never been studied as sole carbon source. In addition, isobutyrate can be a major product of other waste valorization innovations, such as chain elongation (De Leeuw et al. 2020), and microbial electrosynthesis (Vassilev et al. 2018). Here, the aspiration is to produce isobutyrate as a platform chemical. Instead, a mixture of VFAs is produced at low concentrations, resulting in a complex and costly downstream processing (Menon and Lyng 2020). The possibility to produce a solid substance, in the form of a PHA polymer, might facilitate purification and will expand the product spectrum of valorization routes focused on isobutyrate-containing streams. Furthermore, n-butyrate has been identified as the preferred substrate for PHA production due to superior kinetics and product yield. Isobutyrate has an identical theoretical PHA yield (0.94 Cmol/Cmol), if energetically neutral isomerization to n-butyrate is assumed as first conversion step (Shi et al. 1997). Studying the structural isomer of butyrate can give a better understanding of the mechanisms behind this high PHA productivity. Therefore, the aim of this research is to study the suitability of isobutyrate in relation to butyrate as substrate for PHA production with microbial enrichment cultures.

To this end, two sequencing batch reactors (SBRs) were operated with either isobutyrate or butyrate as substrate. For comparison, the operational parameters, which have been successfully applied in studies with acetate, propionate, lactate, and butyrate, were replicated from Johnson et al. (2009a). During the whole enrichment phase the microbial community structure was monitored. At certain points, the performance of the enrichment was characterized, including its maximum PHA storage capacity by way of an accumulation experiment. Additionally, for 1 cycle of the SBR the substrates of both SBRs were exchanged. Finally, the stoichiometric and kinetic parameters of the enrichment in all experiments were derived from a modified version of a metabolic model originally developed by Johnson et al. (2009b).

## Materials and methods

### Enrichment in SBRs

Two double-jacket glass bioreactors with a working volume of 1.4 L (Applikon Biotechnology, The Netherlands) were operated in parallel for the enrichment of a PHA-storing microbial culture on isobutyrate (SBR1) and butyrate (SBR2). The setup and operation of these bioreactors were based on the conditions as described by Johnson et al. (2009a). The bioreactors were operated as non-sterile SBRs, subjected to a feast-famine regime with a cycle length of 12 h and a solids and hydraulic retention time (SRT and HRT) of 24 h, which implies that every cycle 50% of the SBR volume is replaced with fresh medium. The inoculum of the SBRs was aerobic activated sludge of a wastewater treatment plant (AWZI Harnaschpolder Delfluent, The Netherlands). Furthermore, the air flow rate to the bioreactors was set to 0.2 L_N_/min by means of a mass flow controller (MX4/4, DASGIP®, Eppendorf, Germany), and the stirring speed was set to 800 rpm (TC4SC4, DASGIP®, Eppendorf, Germany). The temperature in the bioreactor was controlled at 30 °C ± 0.5 °C with the water jacket around the bioreactor and an external thermostat bath (ECO RE 630 S, Lauda, Germany). The pH was maintained at 7.0 ± 0.1 by the addition of 1 M HCl and 1 M NaOH through an integrated revolution counter (MP8, DASGIP®, Eppendorf, Germany). The pumps for feeding, effluent removal, and pH control, the stirrer, and the airflow were controlled by a hardware abstraction layer (HAL; TU Delft, the Netherlands), which in turn was controlled by a PC using a custom scheduling software (D2I; TU Delft, the Netherlands). The D2I was also used for data acquisition of the online measurements: dissolved oxygen (DO), pH, temperature, acid and base dosage, in- and off-gas composition and feed/water balances. Moreover, the bioreactors were cleaned about twice per week to remove biofilms from the glass walls and the sensors of the bioreactor.

The medium consisted of a carbon and nutrient source with a composition as described by Marang et al. (2013). The carbon source concentration in the SBR was either 9.5 mM isobutyrate or butyrate. The nutrient concentrations in the SBR composed of 6.74 mM NH4Cl, 2.49 mM KH2PO4, 0.55 mM MgSO4·ּH2O, 0.72 mM KCl, 1.5 mL/L trace elements solution according to Vishniac and Santer (1957), and 5 mg/L allylthiourea (to prevent nitrification). To characterize the operational performance, the isobutyrate SBR was subjected three times to a cycle analysis experiment (SBR1-C1 = cycle 51; SBR1-C2 = cycle 111; SBR1-C3 = cycle 115) due to instability of the enrichment, while the butyrate SBR was subjected once to a cycle analysis experiment (SBR2-C = cycle 113).

### Exchange of substrates in SBRs

After the cycle analysis experiments, both SBRs were operated for 1 cycle with exchanged substrate. This means that SBR1 received butyrate as carbon source (SBR1-E = cycle 127), and SBR2 received isobutyrate as carbon source (SBR2-E = cycle 128). During this cycle, the operational performance was characterized. After this cycle the original carbon source was restored.

### PHA accumulation in fed-batch bioreactor

The PHA accumulation experiments were performed in the same bioreactors as the enrichment, but operated in fed-batch mode. The stirring speed, pH, temperature, and aeration rate were kept at the same values as in the SBR. Half of the content of the SBR (700 ml) was used as seeding material for the accumulation experiment. In addition, 700 mL ammonium- and carbon-free medium was supplied. After 30 min, to ensure a temperature of 30 °C, a pulse of 42 mmol isobutyrate or butyrate was supplied to each SBR (SBR1-A = cycle 136; SBR2-A = cycle 138). To prevent carbon source depletion throughout the PHA accumulation, isobutyric acid or butyric acid (1.5 M) and NaOH (1 M) were used to control the pH, ensuring a concentration of 10–30 mM of the carbon source in the bioreactor. Nitrogen was limited during most of the accumulation since no nitrogen source was supplied to the bioreactors and only a small amount (< 0.8 mM of NH_4_^+^) remained from the previous SBR cycle. In this way, growth in the fed-batch bioreactor was limited. If necessary, a few drops of (10 × diluted) Antifoam C (Sigma-Aldrich, USA) were added to inhibit the formation of foam. The experiments were terminated after 11 h.

### Analytical methods

The performance of the cycle, exchange, and accumulation experiments were characterized by online measurements (DO, pH, acid/base dosage, and in-/off-gas composition) with the equipment and software described above, and with offline samples (VFAs, ammonium, PHA, total and volatile suspended solids). The composition of the active biomass was assumed to be CH_1.8_O_0.5_N_0.2_ (Beun et al. 2002). A detailed description of the analytical methods is given by Johnson et al. (2009a). A modification has been made for the ammonium measurement. These samples were measured with a Gallery™ Plus Discrete Analyzer (Thermo-Fisher Scientific, USA).

The method to analyze the PHA composition of the biomass was also described in detail by Johnson et al. (2009a). In brief, the PHA in the biomass was hydrolyzed and esterified in the presence of concentrated HCl, propanol, and dichlorethane with a ratio of 1/4/5 (v/v/v) for 2 h at 100 °C. The formed propylesters, which accumulated in the organic phase, were analyzed by a gas chromatograph (model 6890 N, Agilent, USA). The PHA analysis method was expanded to include the quantification of poly(3-hydroxyisobutyrate) (PHiB) by using methyl (S)-( +)-3-hydroxy-2-methylpropionate (Sigma-Aldrich, USA) as standard.

GC–MS analysis was carried out on a 7890A GC coupled to a 5975C Quadrupole MSD (both from Agilent, USA) to identify PHiB. A detailed description of the analytical protocol is described by Velasco Alvarez et al. (2017).The same sample pre-treatment and the same standard as for GC quantification were used.

### Microbial community analysis

To analyze the microbial composition of the enriched cultures, 2 mL of biomass samples was collected in an Eppendorf tube. The samples were taken 2 times per week, and in addition during the cycle, exchange, and accumulation experiments. The tubes were centrifuged (13,300 g; 5 min.). The pellet was stored at − 20 °C until analysis. After defrosting, genomic DNA was extracted using the DNeasy UltraClean Microbial Kit (Qiagen, Germany), following the manufacturer’s instructions. DNA quantification was carried out using the Qubit® dsDNA Broad Range Assay Kit (Qubit® 2.0 Fluorometer, Thermo Fisher Scientific, USA), following the manufacturer’s instructions. Afterwards, about 50 µL of isolated (16S) DNA was sent to Novogene (China) for amplicon sequencing of the V3-4 region of the 16S rRNA gene. The sequence data have been deposited in GenBank with BioProject ID PRJNA766835.

### Metabolic model and parameter identification

A model, proposed by Johnson et al. (2009b) and adapted towards butyrate by Marang et al. (2013), was used as starting point for this study. This model contains a set of metabolic and kinetic expressions which together describe the consumption and formation of the main compounds in the bioreactor, that is PHA, biomass, organic substrate, CO_2_, O_2_, and ammonium. The trends obtained by the model are fitted to the experimental data.

**Fig. 1:**
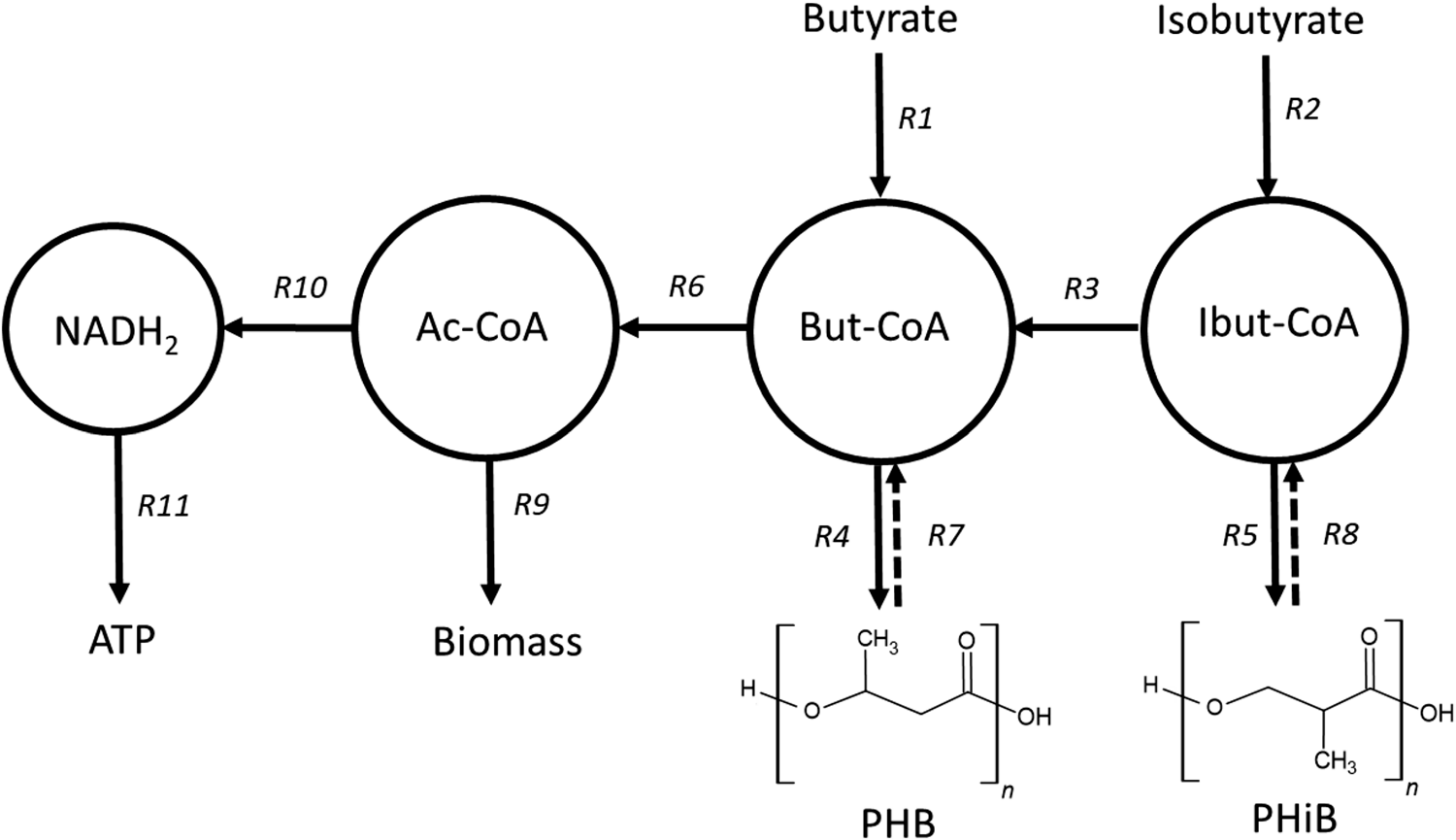
A schematic representation of the proposed PHA metabolism. R1 and R2 represent substrate uptake reactions during the feast phase, depending on the substrate supplied. R3 represents the isomerization of isobutyryl-CoA to butyryl-CoA. R4 and R5 represent the PHB and PHiB production reactions respectively. When isobutyrate is supplied, both PHB and PHiB can be produced. R6 represents the conversion of butyryl-CoA to acetyl-CoA. R7 and R8 represent PHA degradation reactions and are active during the famine phase. R9 and R10 represent anabolic and catabolic reactions respectively. R11 represents the oxidative phosphorylation. Ac-CoA, acetyl-CoA; But-CoA, butyryl-CoA; Ibut-CoA, isobutyryl-CoA

First, an extension of the model was proposed to include isobutyrate and PHiB (see Fig. 1). When feeding with isobutyrate, it was assumed that isobutyryl-CoA was converted to 3-hydroxyisobutyryl-CoA with the enzymes that are also active in valine metabolism (Massey et al. 1976), and then polymerized with a PHA synthase to PHiB (R5 in Fig. 1). At the same time, it was assumed that HB monomers could be formed by isomerization of isobutyryl-CoA to butyryl-CoA (R2, R3, and R4 in Fig. 1). Similarly, it was assumed that PHiB is degraded towards isobutyryl-CoA and isomerized with the same enzyme (R8 and R3 in Fig. 1). In supplementary Table S1, the entire reactions of Fig. 1 are displayed.

The obtained reactions were used to calculate the stoichiometric yields by balancing the conserved moieties (Ac-CoA, But-CoA, IBut-CoA, NADH, ATP). The stoichiometric yields and the kinetic expressions shown in supplementary Table S2 and S3 form the basis of the model. In accordance with the study of Tamis et al. (2014), the PHA degradation function was adapted. For this study, biomass specific rates and actual yields were derived from the model. The efficiency of the oxidative phosphorylation (P/O ratio) was assumed to be 2.0 mol ATP/mol NADH for all experiments (R11 in Fig. 1).

Throughout this work, the terms PHB and PHiB are defined as polymers consisting mainly of HB (3-hydroxybutyrate) monomers or HiB (3-hydroxyisobutyrate) monomers respectively: first, because the data suggest that the micro-organisms in the enrichment produce a mixture of homopolymers rather than a (P (3HB-co-3HiB)) copolymer; second, because this improves the readability of the work. However, it is not excluded that in some of the experiments copolymers or homopolymers with significant amounts of other monomers were produced.

## Results

### Overview enrichment and microbial community characterization

Two SBRs, pulse fed with either isobutyrate or butyrate, were operated for 136 and 138 cycles respectively in this study. The sequencing batch regime was started after the substrate was depleted from the initial batch incubation. SBR1, fed with isobutyrate, had a longer lag phase, and was started when SBR2, fed with butyrate, was operated for 2 cycles already.

**Fig. 2:**
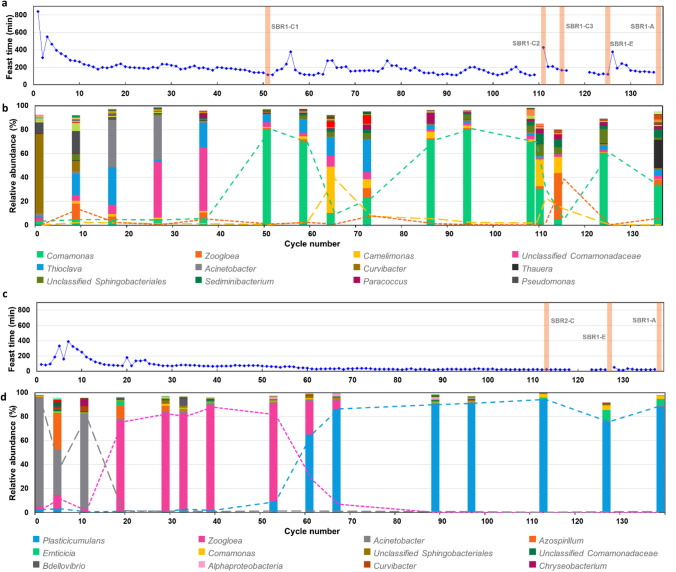
Overview of the feast length and the community structure for the whole enrichment of SBR1 and SBR2. **a** and **c**: feast length of SBR 1 and 2 respectively. The vertical bars represent the different experiments conducted. The gaps in the curve are caused by failure of the data acquisition, not by failure of the SBR performance. **b** and **d**: relative abundance of genera derived from 16S amplicon analysis of SBR 1 and 2 respectively. Only genera reaching a relative abundance higher than 1% are shown in graph. The three most abundant genera are depicted with both stacked columns and lines

The addition of nutrients and carbon source at the beginning of a cycle resulted in a relatively short feast phase, which was succeeded by a relativity long famine phase. The feast phase was characterized by a high oxygen uptake rate, and its duration could therefore be extracted from the DO data (Stouten et al. 2019). In Fig. 2a and c, the duration of the feast phase of SBR1 and SBR2 respectively is plotted for the complete enrichment. Although in both systems a clear downward trend can be observed, the feast time in SBR1 remained at a significantly higher value than SBR2, indicating a low isobutyrate uptake rate and a high butyrate uptake rate. Moreover, the feast length of SBR1 displayed much variability over time, while SBR2 showed a much more stable system in terms of feast length.

The microbial community data, measured by 16S amplicon sequencing, confirms that the stability of the enrichment is low in SBR1 and high in SBR2 (Fig. 2b and c). In SBR1, *Comamonas* sp. is the dominant species, but its presence is highly dynamic. When *Comamonas* sp. becomes abundant, the feast time decreases for most cycles. Its decrease in relative abundance appears to coincide with an increase in feast time and the temporary emergence of *Camelimonas* sp. and *Zoogloea* sp. From the moment that *Comamonas* sp. becomes abundant (cycle 51–135), the average feast time is 167 ± 53 min. SBR2 reveals a very distinct pattern, where 2 stable phases can be distinguished. In the first phase (cycle 27–56), *Zoogloea* sp. is dominant with an average cycle length of 71 ± 7 min, while in the second phase (cycle 62–137) *Plasticicumulans acidivorans* is dominant with an average feast time of 27 ± 5 min.

During the enrichment, the performance of both SBRs was characterized in the form of a cycle analysis experiments, substrate exchange experiments, and accumulation experiments. Figure 2 highlights these key moments in the enrichment and displays the corresponding feast time and microbial community.

### Identification of poly(3-hydroxyisobutyrate)

**Fig. 3:**
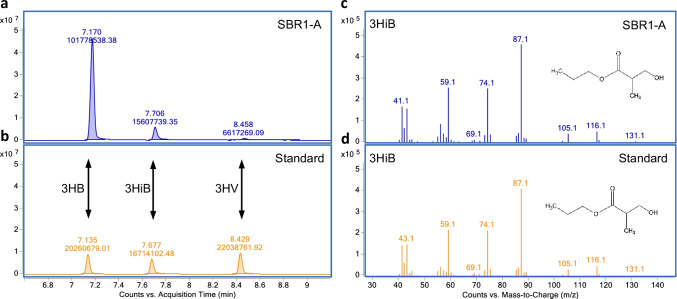
GC–MS analysis of sample from SBR1-A (**a** and **c**) in comparison with standards (**b** and **d**). **a** and **b**: the chromatogram of the GC analysis reveals the three PHA monomers identified in the biomass, that is 3-hydroxybutyrate (3HB), 3-hydroxyisobutyrate (3HiB), and 3-hydroxyvalerate (3HV). **c** and **d**: the mass spectra of the MS analysis reveal a very high degree of similarity of the mass-to-charge ratio of the monomer 3-hydroxyisobutyrate (3HiB), between the sample and the standard

During PHA analysis a peak appeared on the chromatogram which did not match with the propyl ester of 3-hydroxybutyrate or 3-hydroxyvalerate. Nevertheless, its formation and degradation during the SBR cycle resembled a typical PHA shape, a rapid increase during the feast phase and a decrease during the famine phase. Mass spectrometry analysis through GC–MS revealed that the unknown peak had a similar mass spectrum as 3-hydroxybutyrate (data not shown). Then, methyl 3-hydroxy-2-methylpropionate, the methyl ester of the 3-hydroxy variant of isobutyrate, was used as standard. Before analysis, the methyl ester was converted to a propyl ester in the same way as the conventional standard. It appeared that this new standard had the same retention time (Fig. 3a-b) and the same mass spectrum (Fig. 3c–d) as the unknown peak. This demonstrates that the unknown peak represents poly(3-hydroxy-2-methylpropionate), also known as poly(3-hydroxyisobutyrate) (PHiB).

### SBR cycle performance

**Fig. 4:**
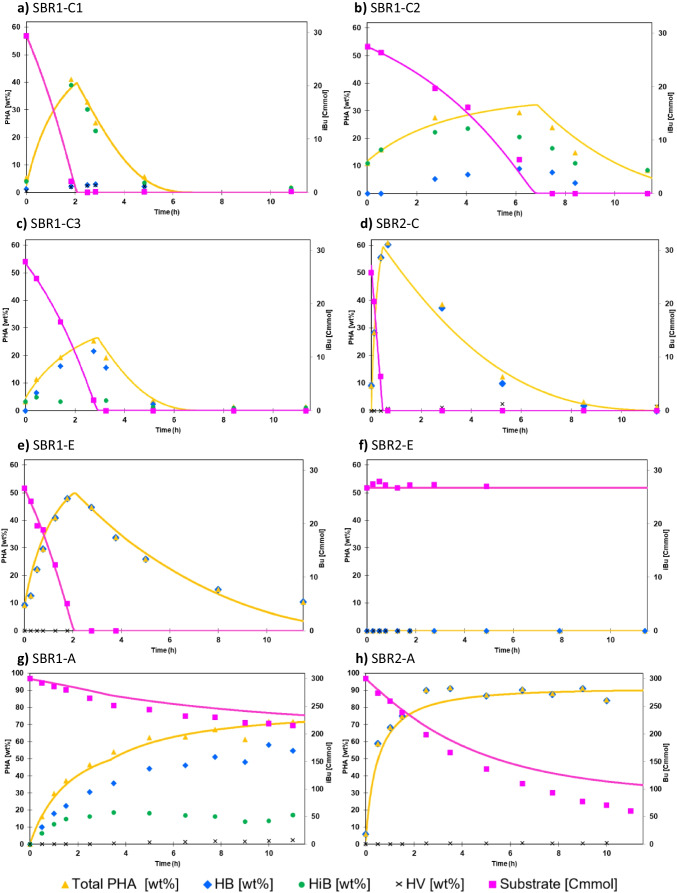
Overview of PHA and substrate analysis of the cycle experiments (**a**–**d**), substrate exchange experiments (**e**–**f**), and accumulation experiments (**g**–**h**). The symbols represent the observed data. Here, the yellow triangles represent the total PHA content, which is a sum of the individual monomers measured (HB, HiB, HV) represented by the remaining symbols. The pink solid line represent the modelled substrate amount in the bioreactor [Cmmol] and the yellow solid line represents the modelled total PHA content [wt%], calculated with Eq. 16 in supplementary Table S3. In the graphs of SBR1 (**a**, **b**, **c**, **e**, **g**), the substrate is isobutyrate; in the graphs of SBR2 (**d**, **f**, **h**), the substrate is butyrate (indicated on secondary axes)

A more detailed insight of the performance of the SBRs was obtained by an extensive analysis of specific operational cycles. For SBR1, multiple cycles were analyzed due to a variation of the functional performance during the enrichment, illustrated by the feast times in Fig. 2a. The main experimental and modelled results of the cycle measurements are depicted in Fig. 4a–d and Table 1. In supplementary Fig. S1, a complete version of the experimental results fitted with the metabolic model is shown, including biomass, ammonium, and off-gas data.

**Table.1:** Overview of the main measured data, microbial community data (16S amplicon sequencing), and model derived yields and biomass specific rates of the different cycle analysis, substrate exchange, and accumulation experiments. *Unc. Sphingo*, unclassified Sphingobacteriales

		Isobutyrate					Butyrate		
	Unit	SBR1-C1	SBR1-C2	SBR3-C3	SRR1-E	SBR1-A	SBR2-C3	SBR2-E	SBR2-A
Type of experiment		Cycle	Cycle	Cycle	Exchange	Accumulation	Cycle	Exchange	Accumulation
**Measured data**									
Length feast phase/time to 60 wt% PHA	h	125	429	166	120	264	25	-	30
PHB max. feast	%	3	9	22	48	58	61	0	91
PHiB max. feast	%	37	24	5	0	16	0	0	0
PHV max. feast	%	3	1	0	0	3	3	0	0
Total PHA max. feast	%	41	32	25	48	72	62	0	92
PHA:X	[gPHB/1gX]	0.7	0.5	0.3	0.9	2.6	1.6	0.0	11.2
**Microbial community data**									
*Comamonas/Plasticicumulans*	(%)	81	30	5	66	35	94	88	76
Other most abundant				*Zoogloea* (39%)					
Second most abundant		*Thioclava* (7%)	*Camelimonas* (23%)	*Camelimonas* (14%)	*Unc. Sphingo.* (13%)	*Thauera* (26%)	*Emticicia* (2%)	*Emticicia* (5%)	*Emticicia* (9%)
**Model derived (feast phase or first 2.5 h of accumulation)**									
q_s,max_	[Cmmol/Cmmol/h]	1.09	0.51	0.74	1.28	0.79	5.48	0.00	2.48
µ_max_	[Cmmol/Cmmol/h]	0.30	0.20	0.31	0.31	0.14	0.02	0.00	0.00
q_PHA,max_	[Cmmol/Cmmol/h]	0.58	0.18	0.20	0.81	0.49	5.15	0.00	2.30
Y_X,S_	[Cmmol/Cmmol]	0.27	0.39	0.41	0.24	0.19	0.00	-	0.00
Y_PHA,S_	[Cmmol/Cmmol]	0.54	0.34	0.34	0.58	0.60	0.96	-	0.92
**Model derived (famine phase)**									
k_d_	[Cmmol^1/3^/Cmmol^1/3^/h]	− 0.80	− 0.40	− 0.75	− 0.27	0.00	− 0.30	0.00	0.00

The first cycle analysis, SBR1-C1, showed the highest substrate uptake rate (feast time is 125 min), the highest relative abundance of *Comamonas* sp. (81%), and the highest PHA content (41.1 wt%) at the end of the feast phase. Surprisingly, the monomer composition of the PHA was strongly dominated by the newly discovered PHiB (37 out of 41 wt%). However, this seemingly competitive functionality appeared to be unstable over the cycles. At certain moments in the enrichment, the community collapses as reflected in the increase in the length of the feast phase, and different dominant strains appear.

The second cycle analysis, SBR1-C2, was conducted at the time of such a collapse. Here, the substrate uptake rate and the PHA production rate of the community dropped abruptly; the feast time becomes 429 min and the q_PHA,max_ is 3.2 times lower than SBR1-C1. Although *Comamonas* sp. is still abundant (30%), a side population of *Camelimonas* sp. appears (23%). This duality is also reflected in the PHA productivity, which is a mixture of PHiB and PHB, as shown in Fig. 4b.

The third cycle analysis, SBR1-C3, was conducted four cycles after the collapse represented by SBR1-C2. According to the feast time, the community had returned to a moderately stable phase, although the feast time was higher than during SBR1-C1 (166 min compared to 125 min). The 16S amplicon sequencing data revealed that the presence of *Comamonas* sp. and *Camelimonas* sp. had decreased and a new dominant species appeared, Zoogloea sp. Figure 4c shows that this well-known PHA producer (Fang et al. 2019; Stouten et al. 2019) was mainly associated with PHB production, rather than with PHiB production, although the PHA content at the end of the feast phase was low (25 wt%).

The contrast of isobutyrate-fed SBR1 with butyrate-fed SBR2 could not be larger. The cycle analysis, SBR2-C, reveals a highly enriched community (94% rel. abundance of *P. acidivorans*), a very high substrate uptake rate (feast time is 25 min), and a very high PHA production rate, as shown in Fig. 4d. In SBR1 a large share of the isobutyrate is used in the feast phase to sustain growth reactions (Y_X,Sfeast_ is 0.27 to 0.41). Butyrate use for growth in the feast is negligible in SBR2-C (Y_X,Sfeast_ is 0.01).

### Substrate exchange

For 1 cycle, the carbon substrates of SBR1 and SBR2 have been exchanged (SBR1-E and SBR2-E). The main results of this experiment are shown in Fig. 4e–f and Table 1. At the time, SBR 1 was dominated by *Comamonas* sp. (rel. abundance of 66%). Therefore, SBR1-E can best be compared to SBR1-C1. It is observed that when SBR1 was fed with butyrate, there was no lag-phase in substrate uptake and the PHA production completely shifted towards PHB production. The total PHA content at the end of the feast phase is slighter higher in SBR1-E (48 wt%) than in SBR1-C1 (41 wt%). The q_s,max_ and q_PHA,max_ have increased slightly, while the other variables remained remarkably comparable. The main difference was that the degradation of PHB in the famine phase is significantly slower in SBR1-E than the degradation of PHiB in SBR1-C1 (k_d_ is − 0.27 and − 0.80 Cmmol^1/3^/Cmmol^1/3^/h respectively).

At the time of the SBR2-E experiment, the SBR2 bioreactor was highly enriched with *P. acidivorans*. Remarkably, this butyrate-enriched community did not possess the capability to metabolize isobutyrate at all. During the 12-h duration of the experiment no substrate, ammonium or O_2_ consumption was observed, and no biomass, PHA, or CO_2_ production was observed (see Fig. 4f and supplementary Fig. S1).

### Maximum PHA accumulation capacity

To evaluate the maximum PHA accumulation capacity, an ammonium limited fed-batch experiment was conducted. Results are shown in Fig. 4g–h and Table 1. At the time, *Comamonas* sp. was not very dominant in SBR1 (rel. abundance of 32%). It seemed that *Thauera* sp., a known PHA producer (Reis et al. 2011; Tamang et al. 2021), had entered the microbial community (rel. abundance of 24%) (Fig. 2b). As in SBR1-C2, the presence of this side population was reflected in the PHA productivity, resulting in a combination of PHiB and PHB. In the first 2 h, the production of both polymer types occurred at a similar rate. Later, it seemed that the production rate of PHiB decreased faster than the production rate of PHB, resulting in a product dominated by PHB at the end of the experiment (55 out of 72 wt%).

SBR2 revealed again a distinct functionality than SBR1. As expected, PHB is the prevailing polymer type. As in the cycle experiments, the PHA production rate and the PHA yield are much more favorable in the butyrate-fed SBR than in the isobutyrate-fed SBR. Additionally, this experiment demonstrates that the PHA accumulation capacity is substantially higher for the microbial community enriched on butyrate than the community enriched on isobutyrate (92 wt% compared to 72 wt%).

The carbon and electron balances of the cycle and exchange experiments closed for 100 ± 3%. For the accumulation experiments, a gap in the mass balance develops, resulting in a closure of 84 ± 3%. This gap has been observed before, and is presumably due to the formation of unknown extracellular compounds over time in accumulation experiments (Marang et al. 2013). In supplementary Table S4, the values for each individual experiment are displayed.

## Discussion

### A new PHA family member

To our knowledge, this is the first scientific article that reports the finding of poly(3-hydroxyisobutyrate). No scientific publications have been found that describe the production of this compound with microbial communities, metabolically engineered organisms, or chemical synthesis. One patent on the production of 3-hydroxyisobutyrate with a metabolically engineered strain mentions poly(3-hydroxyisobutyrate) and proposes the possibility that this polymer is produced by the cultivated bacterium (Marx et al. 2015). However, no measurements were reported to verify this statement. Furthermore, the chemical synthesis of the closely related poly(2-hydroxyisobutyrate) has been described (Pittman et al. 1978; Kricheldorf et al. 2008).

Although PHiB has not been described yet, it is known that environments with a high microbial diversity and a diverse carbon supply have the capacity to produce a broad range of uncommon PHAs. Reports from sewage treatment plants have revealed the presence of PHA consisting of 3-hydroxyhexanoate, 3-hydroxyheptanoate, 3-hydroxyoctanoate, and the branched monomers, 3-hydroxy-2-methylbutyrate and 3-hydroxy-2-methylvalerate (Wallen and Rohwedder 1974; Odham et al. 1986; Queirós et al. 2015). In addition, in the sediments of an estuarine, PHAs consisting of 3-hydroxy-6-methylheptanoic acid and 3-hydroxy-7-methyloctanoate were discovered (Findlay and White 1983). These examples confirm that environmental communities can possess the metabolic capacity to produce uncommon and branched PHAs, and that 3-hydroxy-2-methylpropionate (PHiB) fits this pattern.

### Putative PHiB metabolism

**Fig. 5:**
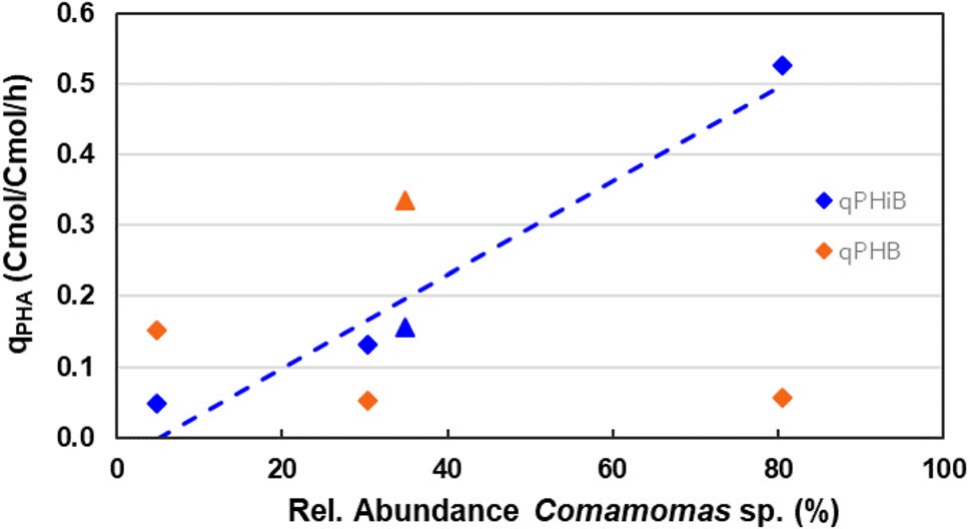
Relative abundance of *Comamonas* sp. versus biomass specific production rate of PHiB and PHB for SBR1-C1 to C3 (  ), and SBR1-A (of first 2.5 h) (  ). The trendline accentuates the correlation between rel. abundance of Comamonas sp. and qPHiB

The presence of *Comamonas* sp. showed a clear correlation with PHiB production (see Fig. 5). PHiB is the dominant polymer when the relative abundance of *Comamonas* sp. is high, and PHB is the dominant polymer when the relative abundance of *Comamonas* sp. has reached a minimum and other species become dominant. Therefore, it is believed that the different micro-organisms in the enrichment produce homopolymers rather than a (P (3HB-co-3HiB)) copolymer.

According to literature, *Comamonas acidivorans* is capable of producing another uncommon PHA consisting completely of 4-hydroxybutyrate (4-HB) monomers, when supplied with the corresponding substrates (Saito and Doi 1994; Lee et al. 2004). This unique feature makes it more plausible that the *Comamonas* sp. found in this study is responsible for the production of PHiB as homopolymer. Moreover, Sudesh et al. (1998) suggests that this competence of *Comamonas acidivorans* was not due to the PHA synthase having a high specificity to incorporate 4-HB monomers, but rather the presence of an efficient metabolic pathway to produce 4-hydroxybutyryl-CoA from related precursors, such as 4-HB and 1,4-butanediol. A comparable principle might be responsible for PHiB production in this study, a *Comamonas* sp. possessing an effective machinery to convert isobutyrate into 3-hydroxyisobutyryl-CoA.

The results of the exchange experiment (SBR1-E) give some additional insights in the metabolic reactions underlying PHiB production. The instant response to this new substrate, butyrate, suggests that the metabolic pathways of PHA production, growth, and catabolism from isobutyrate have overlap with the pathways from butyrate. Moreover, all yields shown in supplementary Table S4 hardly change when the substrate switched from isobutyrate to butyrate. This advocates for the pathway proposed in Fig. 1, because here the carbon and electron stoichiometry is identical, whether PHiB is produced from isobutyrate, PHB from isobutyrate, or PHB from butyrate. In addition, the instant increase of q_s,max_ and q_PHA,max_ in butyrate-fed SBR1-E compared to isobutyrate-fed SBR1-C1, while µ remains very similar, indicates that the uptake of isobutyrate or the production of PHiB is rate-limiting (R2 and R5 in Fig. 1).

The major difference between SBR1-C1 and SBR1-E is the degradation rate of PHiB and PHB in the famine phase respectively, which is slower in SBR1-E than SBR1-C1 (k_d_ is − 0.27 and − 0.80 Cmmol/Cmmol/h respectively). Notably, the k_d_ of SBR1-E is very comparable to the k_d_ of the PHB-producing community of SBR2 (k_d_ is − 0.3 Cmmol/Cmmol/h). These observations make it plausible that a different pathway is used for PHB degradation (R7 in Fig. 1) than for PHiB degradation (R8, R3, and R6 in Fig. 1). This pathway for PHB degradation with acetoacetyl-CoA as intermediate (R7 in Fig. 1 and supplementary Table S1) has been widely accepted in literature as most prevalent pathway (Oeding and Schlegel 1973; Senior and Dawes 1973).

### PHiB production as selective strategy

Pulse-fed sequencing batch reactors with long periods of substrate depletion are well studied systems which are known to select for communities with high substrate uptake rates. Hoarding the substrate in the form of PHA via a small number of enzymatic steps is a productive strategy compared to the complex and relatively slow formation of biomass (Kleerebezem and Loosdrecht 2007). This line of reasoning can be extended to the formation of PHiB compared to PHB when isobutyrate is supplied as carbon source. PHiB is the type of PHA most closely related to isobutyrate, and the formation requires, according to Fig. 1, less enzymatic steps than the formation of PHB, and is, therefore, presumably faster. This idea is confirmed by our results which showed that the smallest feast time (i.e., highest substrate uptake rate) corresponds to the highest fraction of PHiB compared to PHB (e.g., SBR1-C1).

However, our results also showed that possessing the highest substrate uptake rate is not the only factor required to endure as a dominant community. At five moments in the enrichment of SBR1, the feast time increased up to 2- to eightfold of the minimum feast time of 108 min. From two of these events, the community dynamics were captured by 16S amplicon analysis. It revealed that the dominant *Comamonas* sp. washes out as can been seen from the decrease in relative abundance, and other species take over. These opportunistic species appeared rapidly, and disappeared almost with the same pace. Here, the question appears, what caused the fluctuations in community in SBR1?

According to Hibbing et al. (2010), microbial competition for a limiting resource, carbon in our study, can be classified into two different strategies, exploitation and interference competition. Exploitation competition is a passive form of competition, focused on the rapid uptake of the limiting nutrient. Interference competition is an active form of competition, which involves direct antagonistic interaction with the opponent. The data suggest that in SBR2 around the 60th cycle an example of exploitation competition manifested itself. Here, *P. acidivorans* slowly but steadily gained ground against *Zoogloea* sp. until it is washed out. It seems that the competitive strategy of *P. acidivorans* based on very fast substrate uptake coupled to PHA production in absence of growth in the feast phase, is very effective in washing out competitive strains.

The fluctuations in community in SBR1 revealed a very different pattern, and could be explained by interference competition. *Camelimonas* sp. or another species could have secreted an antimicrobial which inhibited the growth of *Comamonas* sp. In addition, there are a range of different microbial interactions difficult to uncover (e.g., viral infections, predation, cross feeding) which may also play a role in the competition of SBR1 (Conthe Calvo 2018).

### Comparison of butyrate and isobutyrate in relation to other carbon sources

**Fig. 6:**
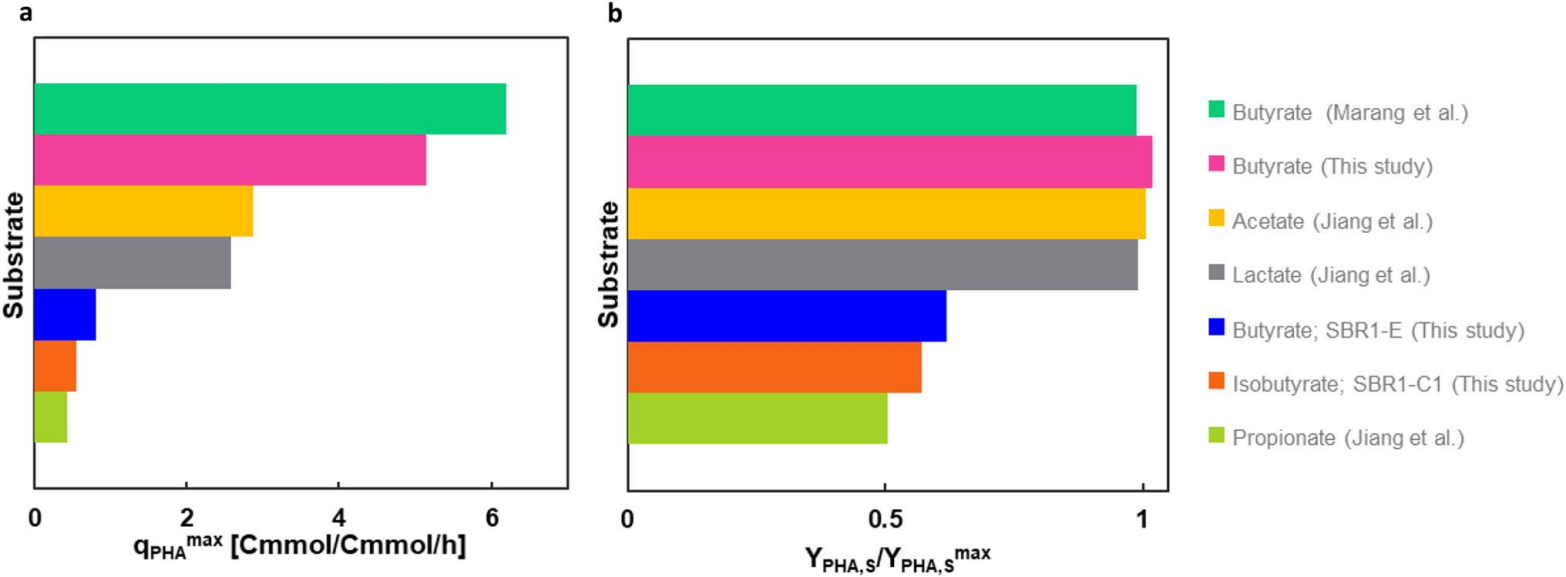
Comparison of this study and other single-carbon studies with virtually identical experimental settings, including acetate and propionate (Jiang et al. 2011a), lactate (Johnson et al. 2009a), and butyrate (Marang et al. 2013). (**a**) q_PHA_^max^ in the feast phase of the SBR cycle. (**b**) The PHA yield as a fraction of the maximal theoretical yield $\left({Y}_{PHA,S}/{Y}_{PHA,S}^{max}\right)$ in the feast of the SBR cycle

The laboratorial research on PHA substrates has reached a level that many different carbon sources (acetate, propionate, lactate, butyrate, isobutyrate) have been assessed on their PHA production potential under virtually identical conditions (Jiang et al. 2011a, b; Marang et al. 2013). All enrichments except isobutyrate were dominated by *Plasticicumulans acidvorans* (acetate, propionate, butyrate), or a close relative (lactate). Butyrate reveals the highest potential in terms of PHA production rate (q_PHA,max_), isobutyrate among the lowest (see Fig. 6a). However, it appears that isobutyrate scores slightly higher than the frequently encountered substrate propionate. Part of these results could be explained by the length of the metabolic pathway from substrate towards PHA, which is for example shorter for butyrate then for acetate, lactate, and propionate. On the other hand, according to Fig. 1, the number of enzymatic steps for isobutyrate and butyrate towards PHA is the same. Therefore, other factors, such as enzyme kinetics, enzyme expression levels, and cell morphology, will also play a role.

Figure 6b displays the PHA yield as a fraction of the maximal theoretical yield as estimated using the metabolic pathways described ${(Y}_{PHA,S}/{Y}_{PHA,S}^{max})$. Here, it appears that butyrate, acetate, and lactate maximize their PHA yield by channelling almost all substrate towards PHA, while minimizing growth. Interestingly, although the propionate enrichment is dominated by *P. acidivorans*, the growth reaction is not eliminated like in the butyrate or acetate enrichments. This would suggest a substrate-related explanation for the poor PHA yield in the propionate enrichment. It is presumed that for propionate and isobutyrate that are both characterized by a lower substrate uptake rate and longer feast phase, a relatively large portion of the substrate is used for growth reactions, and PHA production yields are therefore much lower than the predicted maximum.

On the other hand, when the isobutyrate enrichment is fed with butyrate (SBR1-E), no significant increase is observed of the PHA yield. This would suggest that community-related factors also play a role in the poor PHA yield of the isobutyrate enrichment (i.e., although the enriched community is capable of metabolizing isobutyrate, it is not capable of reaching high PHA yields). When you assume that butyrate or acetate are more abundant in natural environments than isobutyrate, then it could be argued that it makes evolutionary sense to develop a high PHA productivity trait for butyrate and acetate, but not for isobutyrate. The fact that an all-round PHA champion like *P. acidivorans* completely lacks the capability to take up and/or process isobutyrate, as shown in experiment SBR2-E, supports this idea.

### Outlook

The experimental outcomes of structural isomers isobutyrate and butyrate reveal a very distinct behavior in terms of ecological stability, PHA productivity, and PHA composition. Although butyrate is superior from a bioprocess development point of view, this research shows that isobutyrate-rich streams can produce unique PHA polymers, although a lower maximum PHA content can be established in comparison with most other VFAs. This is the first scientific report identifying bacterial PHiB production. It supports the idea that selective environments are a powerful tool to discover new metabolic pathways and new type of polymers. Studying the physicochemical properties of this polymer would be an interesting topic for future research, and might be the onset for a research area to produce PHA specialties by using microbial enrichments.

## Supplementary Information

Below is the link to the electronic supplementary material.

Supplementary file1 (PDF 1519 kb)

## Data Availability

The datasets generated during and/or analyzed during the current study are available from the corresponding author on reasonable request. The sequence data generated during and the current study are available in the GenBank repository under BioProject ID PRJNA766835.
