# Supplementary material for: Production of a newly discovered PHA family member with an isobutyrate-fed enrichment culture
Source: Appl Microbiol Biotechnol. 2022 Jan 5;106(2):605–18. doi: 10.1007/s00253-021-11742-9 (PMC8763783; doi:10.1007/s00253-021-11742-9)
Supplement: Supplementary file 1 — Supplementary file1 (PDF 1519 kb) [file 253_2021_11742_MOESM1_ESM.pdf]

Electronic supplementary material

Applied Microbiology and Biotechnology

Production of a newly discovered PHA family member with an isobutyrate-fed enrichment culture

Chris M. Vermeer[a*], *Larissa J. Bons*[b], *Robbert Kleerebezem*[c]

[a] PhD Candidate, Department of Biotechnology, Delft University of Technology, Van der Maasweg 9, 2629 HZ Delft, The Netherlands
[b] MSc student, Department of Biotechnology, Delft University of Technology, Van der Maasweg 9, 2629 HZ Delft, The Netherlands
[c] Associate professor, Department of Biotechnology, Delft University of Technology, Van der Maasweg 9, 2629 HZ Delft, The Netherlands
*Corresponding author – email address: c.m.vermeer@tudelft.nl, telephone number: +31629174811, fax number: not applicable

Table S1 Metabolic reactions. The metabolic reactions proposed for the isobutyrate and butyrate model are on a carbon-mole base (per Cmol), adapted from Marang et al., 2013. The origin and derivation of the stoichiometric reactions can be found in *Van Aalst-Van Leeuwen et al. (1997). Later the model was optimized by* Johnson et al. (2009), and adapted towards butyrate by Marang et al. (2013). The efficiency of the oxidative phosphorylation (δ) was assumed to be 2.0 for all experiments. But-CoA = Butyryl-CoA, IBut-CoA = Isobutyryl-CoA, Ac-CoA = Acetyl-CoA.

	**Reaction**	**Stoichiometry**
**1.**	Butyrate uptake	1 Cmol But + 0.75 mol ATP → 1 Cmol But-CoA
**2.**	Isobutyrate uptake	1 Cmol IBut + 0.75 mol ATP → 1 Cmol IBut-CoA
**3.**	Isomerase of IBut-CoA to But-CoA	1 Cmol IBut-CoA → 1 Cmol But-CoA
**4.**	PHB production	1 Cmol But-CoA → 1 Cmol PHB + 0.25 mol $NADH_2$
**5.**	PHiB production	1 Cmol IBut-CoA → 1 Cmol PHiB + 0.25 mol $NADH_2$
**6.**	But-CoA to Ac-CoA	1 Cmol But-CoA → 1 Cmol Ac-CoA + 0.5 mol $NADH_2$
**7.**	PHB consumption	1 Cmol PHB + 0.25 mol ATP → 1 Cmol Ac-CoA + 0.25 mol $NADH_2$
**8.**	PHiB consumption	1 Cmol PHiB + 0.25 mol ATP + 0.25 mol $NADH_2$→ 1 Cmol IBut-CoA
**9.**	Growth on Ac-CoA	1.267 Cmol Ac-CoA + 0.2 mol NH_3 + 2.16 mol ATP → 1 Cmol $CH_{1.8}O_{0.5}N_{0.2}$ + 0.267 mol CO_2 + 0.434 mol $NADH_2$
**10.**	Catabolism	1 Cmol Ac-CoA → 1 mol CO_2 + 2 mol $NADH_2$
**11.**	Oxidative phosphorylation	1 Cmol $NADH_2$ + 0.5 mol O_2 → δ mol ATP

Table S2 Overview of stoichiometric yields. In the feast phase, growth, PH(i)B production and maintenance reactions take place, which are driven by substrate consumption. In the famine phase, only growth and maintenance reactions take place, which are driven by PH(i)B consumption. The yields are derived from the metabolic reactions (**Table S1**) and balances for the conserved moieties (NADH, ATP, AcCoA, But-CoA, Ibut-CoA). The method for this derivation is described in detail by Van Aalst-Van Leeuwen et al. (1997). The yields are expressed as a function of the efficiency of the oxidative phosphorylation (P/O ratio, symbol δ). All yields are on carbon-mole base (Cmol/Cmol).

**Feast phase (Butyrate/Isobutyrate)**			
Growth	$Y_{CO_2,X}^{feast,max} = -\frac{40\delta - 291}{250\delta - 75}$	$Y_{O_2,X}^{feast,max} = -\frac{279}{200\delta - 60}$	$Y_{X,S}^{feast,max} = -\frac{250\delta - 75}{210\delta + 216}$
	$Y_{N(Ac),X}^{feast,max} = -0.2$		
PH(i)B production	$Y_{CO_2,PH(i)B}^{feast,max} = -\frac{\delta - 3}{10\delta - 3}$	$Y_{O_2,PH(i)B}^{feast,max} = -\frac{27}{80\delta - 24}$	$Y_{PH(i)B,S}^{feast,max} = -\frac{10\delta - 3}{9\delta}$
Maintenance	$Y_{CO_2,Ac}^{feast,max} = -1$	$Y_{O_2,Ac}^{feast,max} = 1.25$	$Y_{ATP,S}^{feast,max} = 0.75 - 2.5\delta$
**Famine phase (PHB/PHiB)**			
Growth	$Y_{CO_2,X}^{famine,max} = -\frac{15\delta - 241}{225\delta - 25}$	$Y_{O_2,X}^{famine,max} = -\frac{1077}{900\delta - 100}$	$Y_{X,PH(i)B}^{famine,max} = -\frac{225\delta - 25}{210\delta + 216}$
	$Y_{N,X}^{famine,max} = -0.2$		
Maintenance	$Y_{CO_2,PH(i)B}^{famine,max} = -1$	$Y_{O_2,PH(i)B}^{famine,max} = 1.125$	$Y_{ATP,PH(i)B}^{famine,max} = 0.25 - 2.25\delta$

Table S3 Model Kinetics. The kinetic expressions that describe the evolution of most essential compounds in the system. During the feast phase there are six compounds (PHA, substrate, biomass, CO_2, O_2, NH_3). During the famine there is no substrate, so only 5 compounds remain (PHA, substrate, biomass, CO_2, O_2, NH_3). The derivation of the expressions originated in the work of Van Aalst-Van Leeuwen et al. (1997) and Johnson et al. (2009).

**Feast phase**			
PHA production	$q_{PHA,1}^{feast}(t)=\left(q_S(t)-\mu^{feast}(t)\cdot\frac{1}{Y_{X,S}^{feast}}-m_S\right)\cdot Y_{PHA,S}^{feast}$	if $q_{PHA,1}^{feast}\leq q_{PHA,2}^{feast}$	eq.1
With PHA inhibition	$q_{PHA,2}^{feast}(t)=q_{PHA}^{max}\cdot\frac{C_S(t)}{K_S+C_S(t)}\cdot\left[1-(\frac{f_{PHA,X}(t)}{f_{PHA,X}^{max}})^{\alpha}\right]$	if $q_{PHA,1}^{feast}\geq q_{PHA,2}^{feast}$	eq.2
Substrate uptake	$q_{S,1}(t)=q_S^{max}\cdot\frac{C_S(t)}{K_S+C_S(t)}$	if $q_{PHA,1}^{feast}\leq q_{PHA,2}^{feast}$	eq.3
With PHA inhibition	$q_{S,2}(t)=\mu^{feast}(t)\cdot\frac{1}{Y_{X,S}^{feast}}+q_{PHA}^{feast}\cdot\frac{1}{Y_{PHA,S}^{feast}}+m_S$	if $q_{PHA,1}^{feast}\geq q_{PHA,2}^{feast}$	eq.4
Growth	$\mu^{feast}(t)=\mu^{max}\cdot\frac{C_{NH_3}(t)}{K_{NH_3}+C_{NH_3}(t)}\cdot\frac{C_S(t)}{K_S+C_S(t)}$		eq.5
Maintenance	$m_S=\frac{m_{ATP}}{Y_{ATP,S}^{feast}}$		eq.6
CO_2 evolution	$q_{CO_2}^{feast}(t)=\mu^{feast}(t)\cdot Y_{CO_2,X}^{feast}+q_{PHA}^{feast}(t)\cdot Y_{CO_2,PHA}^{feast}+m_S\cdot Y_{CO_2,S}^{feast}$		eq.7
O_2 uptake	$q_{O_2}^{feast}(t)=\mu^{feast}(t)\cdot Y_{O_2,X}^{feast}+q_{PHA}^{feast}(t)\cdot Y_{O_2,PHA}^{feast}+m_S\cdot Y_{O_2,S}^{feast}$		eq.8
NH_3 uptake	$q_{NH_3}^{feast}(t)=\mu^{feast}(t)\cdot Y_{NH_3,X}^{feast}$		eq.9
**Famine phase**			
Growth	$\mu^{famine}(t)=Y_{X,PHA}^{famine}\cdot(q_{PHA}^{famine}(t)-m_{PHA})$		eq.10
PHA degradation	$q_{PHA}^{famine}(t)=k\cdot\left(\frac{C_{x0}}{C_x(t)}\right)^{1/3} f_{PHA,X}(t)^{2/3}$		eq.11
Maintenance	$m_{PHA}=\frac{m_{ATP}}{Y_{ATP,PHA}^{famine}}$		eq.12
CO_2 evolution	$q_{CO_2}^{famine}(t)=\mu^{famine}(t)\cdot Y_{CO_2,X}^{famine}+m_S\cdot Y_{CO_2,PHA}^{famine}$		eq.13
O_2 uptake	$q_{O_2}^{famine}(t)=\mu^{famine}(t)\cdot Y_{O_2,X}^{famine}+m_S\cdot Y_{O_2,PHA}^{famine}$		eq.14
NH_3 uptake	$q_{NH_3}^{famine}(t)=\mu^{famine}\cdot Y_{NH_3,X}^{famine}$		eq.15
**Overall**			
PHA content of cell dry weight	$PHA\ wt\%\ (t)=\frac{c_{PHA}(t)\cdot Mw_{PH(i)B}}{c_{PHA}(t)\cdot Mw_{PH(i)B}+(C_X(t)\cdot Mw_X/(1-\bar{f}_{ash}))}*100\%$		eq.16

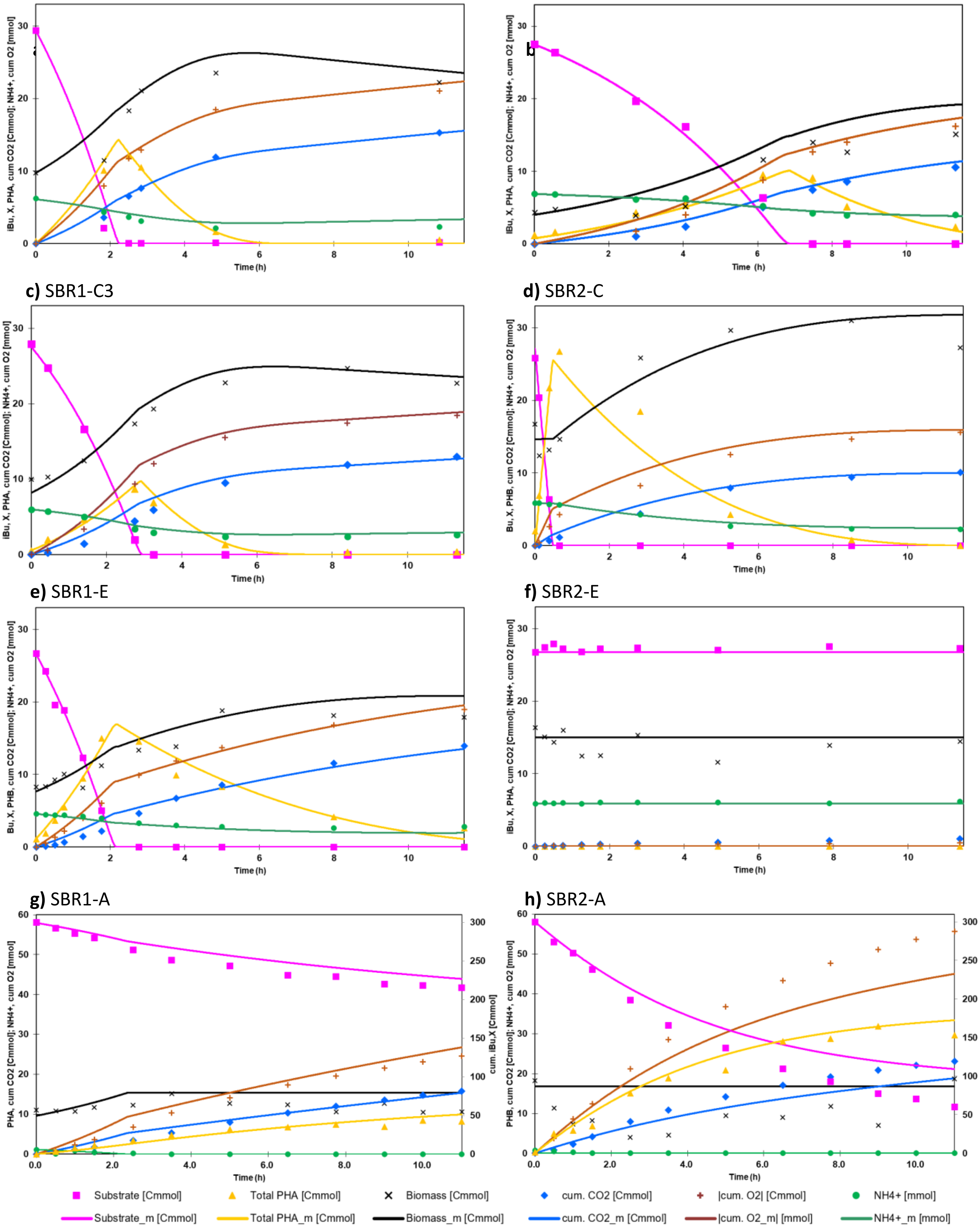

Fig. S1 Modelled and observed results of experiments of the cycle experiments **(a-d)**, substrate exchange experiments **(e-f)**, and accumulation experiments **(g-h)**. The symbols represent the observed data. The solid lines represent the modelled data (indicated with '_m' in legend), calculated with the kinetic expressions depicted in **Table S3**. In the SBR1 graphs (**a, b, c, e, g**) the substrate is isobutyrate, in the SBR2 graphs (**d, f, h**) the substrate is butyrate (indicated on axes). For both CO_2 and O_2 the cumulative values are shown. As O_2 is consumed, the absolute value of the cumulative values is depicted.

Table S4 Outcome of the carbon and electron balance analysis, additional model derived yields, and input parameters of the model. The carbon balances and the electron (degree of reduction) balances were set up for all 7 experiments as an indication of the accuracy of the measurements. Here, the flux of carbon and electrons into the system was subtracted from the flux out of the system. mATP represents the amount of ATP required per Cmol biomass per unit of time, and was fitted to the data for every individual experiment. The P/O ratio reflects the efficiency of the oxidative phosphorylation and was assumed to be 2 mol ATP/mol NADH for all experiments (Beun et al. 2000).

	Unit	Isobutyrate					Butyrate		
		SBR1-C1	SBR1-C2	SBR3-C3	SRR1-E	SBR1-A	SBR2-C3	SBR2-E	SBR2-A
**Type of experiment**		Cycle	Cycle	Cycle	Exchange	Accumulation	Cycle	Exchange	Accumulation
**Carbon and electron balance**									
C-Balance	%	2.33	2.63	2.23	-3.04	-11.92	-4.01	-0.47	-19.95
e-balance	%	1.15	2.58	2.09	-2.01	-13.80	-5.13	-0.38	-17.77
**Model derived yields (first 2.5h of accumulation)**									
Y_{X,S_feast}	[Cmmol/Cmmol]	0.27	0.39	0.41	0.24	0.19	0.004	-	0.00
$Y_{PHA,Sfeast}$	[Cmmol/Cmmol]	0.54	0.34	0.34	0.58	0.60	0.96	-	0.92
Y_{CO2,S_feast}	[Cmmol/Cmmol]	0.19	0.26	0.25	0.18	0.21	0.06	-	0.08
Y_{O2,S_feast}	[mmol/Cmmol]	0.37	0.45	0.44	0.35	0.37	0.19	-	0.21
Y_{X,PHA_famine}	[Cmmol/Cmmol]	0.56	0.52	0.53	0.44	-	0.67	-	-
Y_{CO2,PHA_famine}	[Cmmol/Cmmol]	0.63	0.48	0.73	0.56	-	0.33	-	-
**Input parameters model**									
mATP (fitted)	mmol/Cmmol/h	0.11	0.10	0.08	0.13	0.25	0.00	0.00	0.20
P/O ratio	-	2.00	2.00	2.00	2.00	2.00	2.00	2.00	2.00
